# Functional Chitosan-Based Composite Film Incorporated with 3-(Methylthio) Propyl Isothiocyanate/α-Cyclodextrin Inclusion Complex for Chicken Meat Preservation

**DOI:** 10.3390/polym14214655

**Published:** 2022-11-01

**Authors:** Hongyan Wu, Xinying Ao, Jianan Liu, Junya Zhu, Jingran Bi, Hongman Hou, Hongshun Hao, Gongliang Zhang

**Affiliations:** 1 School of Food Science and Technology, Dalian Polytechnic University, Dalian 116034, China; 2 Liaoning Key Laboratory for Aquatic Processing Quality and Safety, Dalian 116034, China; 3 Jinkui Food Science and Technology Corporation, Dalian 116033, China; 4 Department of Inorganic Nonmetallic Materials Engineering, Dalian Polytechnic University, Dalian 116034, China

**Keywords:** 3-(methylthio) propyl isothiocyanate, α-cyclodextrin, chitosan, inclusion complex, composite film, antibacterial activity, food packaging

## Abstract

The 3-(Methylthio) propyl isothiocyanate (MTPITC)-loaded inclusion complex prepared by α-cyclodextrin (α-CD) was incorporated into chitosan (CS) film to fabricate a packaging material for fresh chicken meat preservation. Scanning electron microscope images indicated homogenous dispersion of the MTPITC-α-CD in CS polymer. Fourier-transform infrared and X-ray diffraction techniques revealed that MTPITC-α-CD was incorporated into the CS film matrix by the physical interactions. The introduction of MTPITC-α-CD improved the UV-vis light-blocking ability, with a slight loss of transparency. Although the water solubility and water vapor barrier capacity were not significantly influenced by the addition of MTPITC-α-CD, the antioxidant attribute was significantly enhanced. The CS-MTPITC-α-CD film displayed obvious and sustained suppressive effects against *Salmonella typhimurium*, with the inhibition zone diameters of 14.7 mm at 12 h and 7.3 mm at 24 h, respectively. Moreover, the quality index analysis indicated that the CS-MTPITC-α-CD film-wrapped fresh chicken, during refrigerated storage, exhibited better preservative efficacy than the control groups, with the total viable counts of 6.5 Log CFU/g, total volatile base nitrogen of 8.4 mg/100 g, pH of 6.6, thiobarbituric acid-reactive substances of 0.2 mg/kg, and the sensory score of 5 at day 16. Collectively, these results suggest that CS-MTPITC-α-CD film is a prospective packaging candidate for delaying the quality deterioration of chicken meat.

## 1. Introduction

In recent decades, to fight or alleviate food-borne bacteria-caused illnesses, designing and developing foodstuff packaging materials possessing antimicrobial and preservative potential have risen in appeal among increasingly growing health concerns [1,2]. Meanwhile, with the long-standing environmental problems posed by the application and disposal of petrochemical materials, the eco-friendly and sustainable nature-derived biopolymers, such as chitosan (CS), yam starch, and pectin, have become prospective packaging material candidates [3,4,5]. CS, one of the most ubiquitously present biopolymers in nature [6], has been widely reported as an edible film because of its superb biocompatibility, film-forming property, and oxygen barrier ability [7]. However, the inherent antibacterial and antioxidant efficacies of pure CS film are still restricted, which motivates the research on fortifying the plain film matrix with nature-derived active compounds [8].

Natural bioactive substances originating from plants, for instance, organosulfur compounds [9], polyphenolic compounds [10], and vitamin E [11], have been incorporated into the CS-based biopolymers to attribute the composite materials’ enhanced physicochemical and functional properties. For example, the addition of quaternized cellulose nanocrystals-coated benzyl isothiocyanate improved the mechanical performance as well as the barrier capacity of the composite film composed of CS and cellulose nanofibers, endowing the film with excellent antimicrobial potential, and thereby benefiting the preservation of chicken meat [12]. In addition, recent evidence has revealed that adding the sulfur flavor molecule, allyl isothiocyanate (AITC), to the CS/carrageenan coating efficiently reduced the proliferation of *Salmonella* Typhimurium and *Campylobacter coli* inoculated on the chicken meat during low-temperature storage [13]. 3-(Methylthio) propyl isothiocyanate (MTPITC) is another important sulfur-containing compound with a volatile property [14], derived from plant glucoiberverin degradation [15], and has been demonstrated to exhibit potent antimicrobial efficacy against a variety of foodborne and spoilage microbes [16]. Mechanism research has suggested that MTPITC displayed antibacterial potential against *Listeria monocytogenes* by reducing the mRNA levels of the virulence-related proteins and attenuating the biofilm formation [17]. In addition, MTPITC could decrease the transcriptional level of the extracellular protease in *Hafnia alvei* H4 by regulating the gene expression of quorum sensing-associated protein [18]. Hence, these findings led us to suppose that incorporating MTPITC into the CS-based film matrix as the antibacterial or preservative agent might be a feasible scheme to prolong the storage period of perishable foodstuffs. Despite the antibacterial merit of MTPITC, the practical application of this compound still has some apparent drawbacks, such as the strong volatility and inferior aqueous solubility. To solve these drawbacks, encapsulation of MTPITC seems to be a workable method for protecting its bioactive property. Alpha-cyclodextrin (α-CD), one type of the cyclic oligosaccharides, is capable of forming an inclusion complex by encapsulating hydrophobic bioactive substances into its lipophilic cavity [19]. Previous studies indicated that trapping AITC into the α-CD cavity was beneficial in improving solubility in aqueous environments and sustaining the release of this highly volatile compound [20,21]. Moreover, α-CD-encapsulated moringin isothiocyanate not only effectively increased its poor bioavailability, but also triggered neuronal protective signaling pathways to facilitate neuronal repair, which in turn promoted the therapeutic efficacy of this compound [22].

In this study, the MTPITC-loaded inclusion complex prepared by α-CD was added to the CS polymeric chain to prepare a novel food packaging material. The impacts of the MTPITC-α-CD on the microstructure, UV-vis light shielding capacity, moisture barrier performance, and antioxidant and antibacterial capacities of the CS film matrix were comprehensively explored. Moreover, the practical application potential was assessed by examining the preservative efficacy of CS-MTPITC-α-CD film on fresh chicken meat based on the quality index analysis.

## 2. Materials and Methods

### 2.1. Materials

α-CD (purity higher than 98%) and the aliphatic isothiocyanate MTPITC (purity higher than 99%) were procured from Sigma-Aldrich (St. Louis, MO, USA). CS (degree of acetylation: higher than 95%) was purchased from Macklin Biochemical Co., Ltd. (Shanghai, China). *Salmonella enterica* serovar *Typhimurium* (*S. typhimurium*, ATCC 14028) was obtained from the Culture Collection (Beijing, China). The chicken meat was purchased from the local supermarket (Dalian, China). Other reagents utilized in the present study were all of the analytic grades acquired from Aladdin Biochemical Technology Co., Ltd. (Shanghai, China) or Tianjin Damao Chemical Co., Ltd. (Tianjin, China).

### 2.2. Preparation of the MTPITC-α-CD Inclusion Complex

According to the best encapsulation efficiency ratio attained in the initial assays, the molar ratio of MTPITC and α-CD was prepared as 1:2 and the magnetic stirring temperature was 65 °C. Briefly, 10.3 mM of MTPITC anhydrous ethanol solution was mixed with 20.6 mM of α-CD aqueous solution for further stirring for three hours. The resultant solution was treated by ultrasonic wave for 30 min and then freeze-dried.

### 2.3. Preparation of the CS-MTPITC-α-CD Film

The CS-MTPITC-α-CD film was prepared based on our previously reported method [23], in which the pure CS film was used as the control. Briefly, 2% (*w/v*) CS powder was added to 1% (*v/v*) of acetic acid solution and stirred at 60 °C for 1 h. Following this, 1% (*w/v*) of the inclusion complex was added to the CS aqueous solution and stirred for 30 min, and then 2.5% (*v/v*) of glycerol was added while further magnetic stirring for 30 min occurred. The mixture was ultrasonically treated for 2 h before pouring into the mold for casting. The poured solution was dried at 40 °C for 6 h and subsequently conditioned at ambient temperature for two days before use.

### 2.4. Structural Characterization of the MTPITC-α-CD Inclusion Complex and the CS-MTPITC-α-CD Film

#### 2.4.1. FTIR Analysis

The FTIR spectra of α-CD, MTPITC-α-CD, CS, and CS-MTPITC-α-CD were measured by the FTIR instrument (Norwalk, CT, USA). Each spectrum was carried out from 650 cm^−1^ to 4000 cm^−1^ with a resolution of 4 cm^−1^.

#### 2.4.2. XRD Analysis

The XRD patterns of α-CD, MTPITC-α-CD, CS, and CS-MTPITC-α-CD were acquired by the X-ray diffractometer (Shimadzu, Kyoto, Japan). The instrument provided a Cu-Κα radiation source with a voltage of 40 kV. The analysis was carried out at the 2θ scan ranging from 10° to 60°.

### 2.5. Characterization of the CS-MTPITC-α-CD Film

#### 2.5.1. Surface Morphology

The surface microstructure of CS and CS-MTPITC-α-CD films was observed by scanning electron microscopy (SEM) (Hitachi, Tokyo, Japan) at a magnification of ×100.

#### 2.5.2. Light Transmittance

The light transmittance spectrum of CS and CS-MTPITC-α-CD films were tested by the UV-vis spectrophotometer (Cambridge, MA, USA) with the scan ranging from 200 nm to 800 nm.

#### 2.5.3. Water Solubility (WS)

The test film samples were clipped into the 10 mm × 20 mm shape and then placed at 105 °C for one day until obtaining a constant weight, recorded as W_0_. Subsequently, the test film was immersed in 50 mL of distilled water for one day. After removing the excess water, the film was placed at 105 °C for one day and then achieved a constant weight (W_1_) [24]. The WS value was computed using the following formula:

WS (%) = (W_0_ − W_1_)/W_0_ × 100 (1)

#### 2.5.4. Water Vaper Permeability (WVP)

The WVP values of the test film samples were measured by our previously reported method [23]. Briefly, a tailor-made vessel with an inner diameter of 4 cm and a height of 2.5 cm was utilized. The vessel was added with three grams of anhydrous calcium chloride, covered with the test film sample, and then transferred to a desiccator with a 75% room humidity. The weight of the vessel was measured every 24 h during the 72 h of the monitoring period. The WVP (g/m·s·Pa) of the test film was calculated using the following formula:

(2) WVP (g/m·s·Pa) = (Δm × L)/(A × Δt × ΔP)

where Δm represents the weight variation (g) of the test vessel, L  is the thickness (m) of the sample, A  is the permeation area (m^2^), Δt  is the storage time (s), and ΔP represents the vapor water pressure difference of the inside and outside surface of the test film.

#### 2.5.5. Antioxidant Property

The antioxidant capacity of CS and CS-MTPITC-α-CD films were investigated utilizing the DPPH free radical scavenging assay based on our previously reported method [23]. In brief, 100 mg of the film sample was added to 10 mL of ethanol to magnetic stirring at ambient temperature for 24 h to acquire the film extract solution. Following this, 3 mL of the film extract solution was quickly mixed with an equal volume of 0.1 mL of DPPH ethanol solution. The obtained mixture was placed in the dark for 30 min and then used to determine the absorbance at 517 nm, and the DPPH scavenging ability was calculated using the following formula:

DPPH scavenging activity (%) = (A_blank_ − A_sample_)/A_blank_ × 100 (3)

where A_blank_ and A_sample_ mean the absorbance of the reaction solution without and with the test film sample, respectively.

#### 2.5.6. Antibacterial Property

The antibacterial property of the test film samples was examined according to Jiang et al. [12], with minor modifications. Briefly, *S. typhimurium* strain ATCC14028 was cultured in Luria-Bertani (LB) medium overnight at 37 °C to reach the logarithmic phase. Following this,100 μL of the diluted bacterial suspension (approximately 1 × 10^5^ CFU/mL) was spread on the LB agar medium. Afterwards, the well-cut film sample (with a diameter of 6 mm) was added to the agar plate. The antibacterial activity of the film samples was investigated by measuring the diameters of the inhibition zones after 12 h and 24 h of co-incubation.

### 2.6. Application of the CS-MTPITC-α-CD Film on Fresh Chicken Meat

#### 2.6.1. Fresh Chicken Meat Treatments

To evaluate the preservative application potential of the CS-MTPITC-α-CD film on the fresh chicken meat at 4 °C, the variations in the quality characteristics during 16 d of storage were examined. Five grams of fresh chicken meat cubes was wrapped with identical sizes (5 × 5 cm pieces) of polyethylene terephthalate (PET) film, CS film, or CS-MTPITC-α-CD composite film and vacuum-sealed by a vacuum sealer. The quality index analysis was performed at a 4-day interval and the commercially used PET film was considered as the control.

#### 2.6.2. Total Viable Count (TVC) Quantification

At each indicated monitoring time point, the chicken meat samples were homogenized with 45 mL of sterilized normal saline at 10,000 rpm for 60 s. Following this, 0.1 milliliter of suitable gradient-diluted mixture was spread on the plate for the microbial colony counting.

#### 2.6.3. Total Volatile Base Nitrogen (TVB-N) Measurement

On each sampling day, chicken meat samples were homogenized with 25 mL of distilled water for 30 min. After filtration, five milliliters of the supernatants were alkalinized with an equal volume of 10 g/L of magnesium oxide, followed by distilling for five minutes. The volatile nitrogenous substances were absorbed by 20 g/L of boric acid solution and titrated by 0.01 M of hydrochloric acid standard solution until the color turned to bluish violet [25]. The TVB-N of the chicken meat was expressed as mg/100 g of chicken meat sample.

#### 2.6.4. pH Measurement

The chicken meat samples at the indicated monitoring time points were cut into small pieces and completely homogenized in 45 mL of deionized water for 2 min. The mixture was filtered, and the pH of the supernatant was determined [7].

#### 2.6.5. Thiobarbituric Acid–Reactive Substances (TBARS) Measurement

On each sampling day, 0.2 g of the test chicken samples was added to the 25 mL volumetric flask, dissolved with 10 mL of 1-butanol, and diluted to scale. Five milliliters of the mixture were thoroughly mixed with an equal volume of thiobarbituric acid and then heated at 95 °C for two hours. The resultant solutions were then cooled to the ambient temperature and the absorbance at 532 nm was measured [26]. The TBARS values were expressed as mg/kg of the chicken meat sample.

#### 2.6.6. Sensory Evaluation

The organoleptic analysis of all test meat samples was performed by six trained assessors from Dalian Polytechnic University (Dalian, China) using a 1-10 hedonic scale (10 = best quality, 5 = acceptable limit, 1 = worst quality) [27]. The sensory attributes of the test chicken meat were evaluated based on appearance, color, viscosity, odor, texture, and overall acceptability.

### 2.7. Statistical Analysis

All tests were carried out at least in triplicate and all results were presented as mean ± standard deviation. The statistical analysis was carried out by a one-way analysis of variance followed by Duncan’s multiple comparison test or Student’s paired two-tailed t-test (*p* < 0.05) using the SPSS version 16.0 (IBM, Chicago, IL, USA).

## 3. Results and Discussion

### 3.1. Structural Characterization of the MTPITC-α-CD Inclusion Complex and CS-MTPITC-α-CD Film

**Figure 1:**
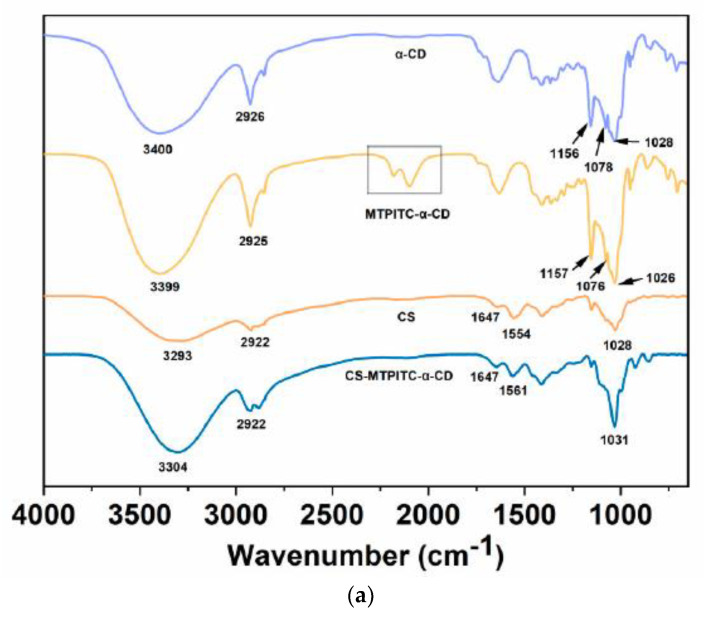

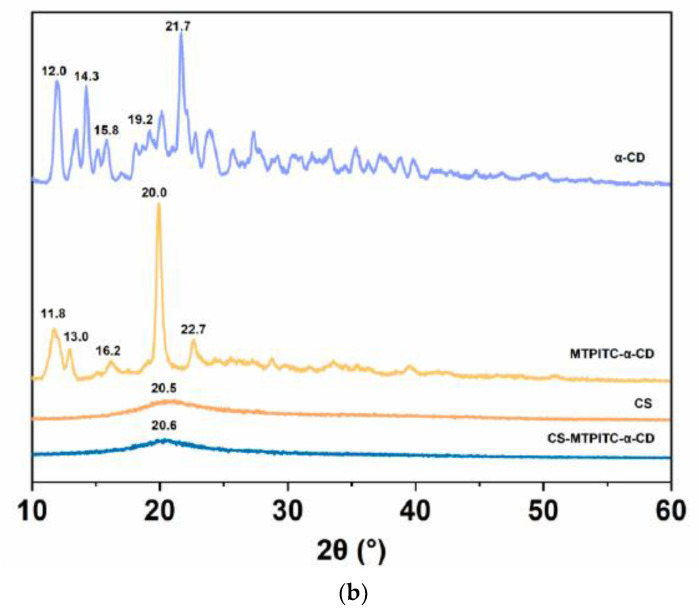
Structural characterization of the MTPITC−α−CD inclusion complex and the CS−MTPITC−α−CD film. (**a**) FTIR spectra of α−CD, MTPITC−α−CD, CS, and CS−MTPITC−α−CD; (**b**) XRD patterns of α−CD, MTPITC−α−CD, CS, and CS−MTPITC−α−CD.

The analysis of FTIR spectra is a powerful manner to reveal the intermolecular interactions among the film components by providing information about characteristic peaks and intensity changes [28]. In Figure 1a, the feature absorption bands of α-CD appear at 3400 cm^−1^, 2926 cm^−1^, 1156 cm^−1^, 1078 cm^−1^, and 1028 cm^−1^, which belonged to the O-H symmetric stretching vibration, C-H stretching vibration, C-O-C glycosidic bridge stretching vibration, and C-O/C-C stretching vibrations, respectively [29,30]. The FTIR spectrum of pure MTPITC has been illustrated in our recent report, in which the prominent peak, displayed at around 2140–2040 cm^−1^, corresponded to the thiocyanate group (N=C=S) [31]. In the current study, after being encompassed in the α-CD, the characteristic absorption peaks of MTPITC at 2140–2040 cm^−1^ were still observed, suggesting that MTPITC exists in the inclusion complex. Moreover, in line with other reported CD-composed inclusion complexes [32,33], the feature absorption peaks of MTPITC-α-CD slightly shifted in comparison with the pure α-CD, indicating that intermolecular interactions might be generated between MTPITC and α-CD. On the other hand, the feature peaks of pure CS exhibited at 3293 cm^−1^, 2922 cm^−1^, 1647 cm^−1^, 1554 cm^−1^, and 1028 cm^−1^, which corresponded to the stretching vibrations of the N-H group and O-H group, asymmetric and symmetric vibrations of the C-H group, amide I band, amide II band, and C-O stretching vibration, respectively [34,35,36]. Notably, some of these characteristic absorption peaks displayed slight shifts and intensity changes with the addition of MTPITC-α-CD. The N-H and O-H groups, as an example, varied from 3293 cm^−1^ to 3304 cm^−1^, which suggested that certain hydrogen bonds in the CS polymeric chain were destroyed due to the introduction of MTPITC-α-CD [37]. In addition, the peak assigned to the N-H and O-H groups in the CS-MTPITC-α-CD film became sharper, possibly relevant to the overlapping of the O-H stretching vibration in the MTPITC-α-CD [32]. Moreover, the amide II band slightly shifted from 1554 cm^−1^ to 1561 cm^−1^, presumably due to the hydrogen bonds formed between CS and MTPITC-α-CD leading to the reduction of hydrophilic groups in the CS polymer chain [33]. Collectively, given that the basic FTIR fingerprint of CS was not significantly influenced by the introduction of MTPITC-α-CD, this information supports the reasonable conclusion that MTPITC-α-CD is embedded into the CS film matrix by physical forces, such as the hydrogen bonds.

XRD is proposed as a useful approach to determining crystal states of the test substances, in which the new generations or changes of characteristic peaks represent the formation of the complex [38,39]. Figure 1b presents the XRD spectra of α-CD, MTPITC-α-CD, CS, and CS-MTPITC-α-CD. In agreement with the previous study, α-CD presented sharp and intense peaks at 2θ values of approximately 12.0°, 14.3°, 15.8°, 19.2°, and 21.7°, reflecting the highly crystalline status of this compound [28]. The inclusion complex was confirmed due to the comparatively broad peaks at other positions (2θ of 11.8°, 13.0°, 16.2°, 20.0°, and 22.7°) observed for MTPITC-α-CD. Furthermore, the CS and CS-MTPITC-α-CD films only displayed a prominent peak at 2θ values of around 20°, corresponding to the crystal lattice of the CS film matrix [40]. Moreover, the feature diffraction peaks of MTPITC-α-CD disappeared, and no new peaks were observed after its incorporation into the CS, suggesting that MTPITC-α-CD was completely embedded in the CS polymer chain by physical interactions.

### 3.2. Physical Appearance, Surface Morphology, and Light Transmittance of CS and CS-MTPITC-α-CD Films

**Figure 2:**
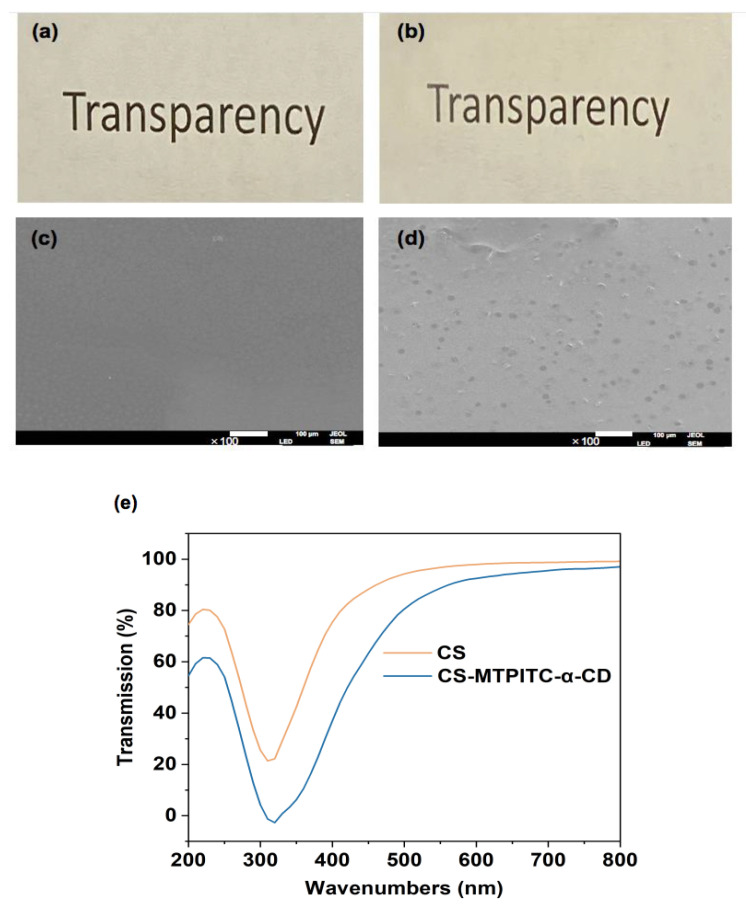
Physical appearance and surface morphology of the CS and CS−MTPITC−α−CD films. Physical appearance of (**a**) CS and (**b**) CS−MTPITC−α−CD films; surface morphology of (**c**) CS and (**d**) CS−MTPITC−α−CD films; (**e**) UV–vis light transmittance of CS and CS−MTPITC−α−CD films.

The physical appearance of packaging materials might directly influence consumers’ choices of foodstuffs; therefore, the macro-morphology of the CS-MTPITC-α-CD was initially investigated. As presented in Figure 2a,b, the CS and CS-MTPITC-α-CD films were both transparent with a slightly yellowish color, plausibly indicating that the addition of MTPITC-α-CD imposed little effect on the physical appearance of the pure CS film. To further evaluate the effects of MTPITC-α-CD on the CS polymer matrix, the surface morphology was captured by SEM (Figure 2c,d). The pure CS film presented a smooth and continuous surface, without the appearance of pores or cracks. By contrast, the surface of the composite film presented several homogeneous granules, suggesting the favorable compatibility between MTPITC-α-CD and CS. A recent study revealed that the direct addition of cinnamon essential oil stabilized with ethyl-N^α^-lauroyl-L-arginate hydrochloride to the CS film matrix could have brought about the holes in the well-organized microstructure of CS molecules. However, the additional introduction of hydroxypropyl-β-cyclodextrin presented a much smoother CS film surface, with smaller-sized holes, because its encapsulation efficacy remarkably enhanced the compatibility among multiple film components [41]. Resemble surficial morphology was reported in other studies fabricating CS-based films with CD-encapsulated bioactive ingredients [42,43].

The UV and visible light exposure have been reported to exacerbate the oxidation of nutritional components in foodstuffs and lead to an off-flavor [44]. Thus, it is imperative to exploit novel food packaging films with excellent light-blocking properties. As displayed in Figure 2e, the percentage transmittance of the pure CS film declined with the addition of MTPITC-α-CD at the light wavelength ranging from 200 nm to 800 nm. Notably, at the UV spectra range (200–400 nm), the CS-MTPITC-α-CD film displayed a significantly reduced UV light transmittance in comparison with the CS film. This indicated that the introduction of MTPITC-α-CD effectively enhanced the UV light barrier capacity of CS film, which might be ascribed to the light reflection and scattering features of the added inclusion complex [43]. Cui et al. [45] proved that the incorporation of cinnamaldehyde-loaded montmorillonite particles increased the UV light-blocking ability of the CS film through the reflection and scattering of UV. Lu et al. [46] prepared a composite film by adding hexahydro-β-acids-2-O-methyl-β-cyclodextrin inclusion to the CS film and also revealed a remarkable reduction of UV light transmittance. In addition, combined with Figure 2b, it was still possible to observe the letters covered by the CS-MTPITC-α-CD composite film. Collectively, these data demonstrated that the CS-MTPITC-α-CD film with sufficient transparency had a promising prospect as the packaging material against UV-vis light irradiation, which improved the prevention of light exposure-induced oxidation in the food system.

### 3.3. WS, WVP, and Antioxidant Properties of CS and CS-MTPITC-α-CD Films

**Figure 3:**
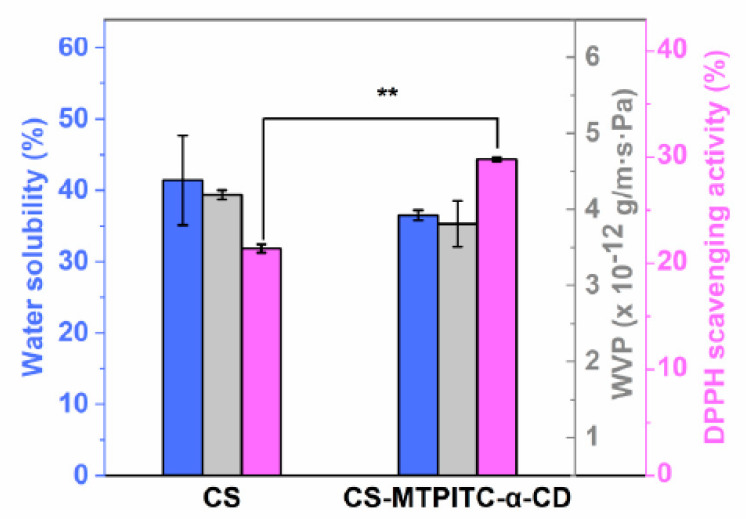
Water solubility (WS), water vapor permeability (WVP), and antioxidant capacity of CS and CS−MTPITC−α−CD films. ** *p* < 0.01 compared to the pure CS film group. Blue bars indicate WS, gray bars indicate WVP, and pink bars indicate DPPH scavenging activity.

The WS level is the foremost parameter reflecting the resistance of the test film against water molecules [47]. As displayed in Figure 3, the WS value of the pure CS film was slightly decreased with the introduction of MTPITC-α-CD. This could be assigned to the hydrogen bondings between the hydroxyl or amino groups of the CS film and the hydrophilic groups of MTPITC-α-CD (Figure 1). A previous study, which fabricated a composite film containing curcumin, also suggested that the addition of an active compound caused negligible variations to the WS value of the CS film matrix [48].

WVP is another critical index for evaluating the barrier capacity of food packaging materials towards moisture [49]. The change in WVP value is usually related to the solubility and diffusion of water molecules in the film matrix [50]. As displayed in Figure 3, the addition of MTPITC-α-CD did not cause a significant decrease in the WVP index of the pure CS film, which might be ascribed to two aspects. On one hand, non-significant WS variations were observed between the CS film and the CS-MTPITC-α-CD film (Figure 3), suggesting that the moisture hydrophilic nature of CS film was not remarkably affected by the incorporation of MTPITC-α-CD. Correspondingly, the WVP index of the hydrophilic matrix, which reflects the interaction between the moisture molecules and film matrix [51], showed a non-significant decrease. On the other hand, the high-density nature of the CS film matrix might also be associated with the negligible variations of CS and CS-MTPITC-α-CD films [23]. Taken together, these findings indicated that the CS film matrix might be a predominant factor affecting WVP value changes. Similar insignificant reductions of WVP were also reported in the CS-based films enriched with benzyl isothiocyanate (BITC)-α-CD inclusion complex [23] and quaternized cellulose nanocrystals-coated BITC [12].

Oxidation induced by free radicals is an important issue during fresh foodstuff storage because the oxidative process would pose undesirable effects on the food, including discoloration, off-flavors, nutrition, and quality losses [52]. As illustrated in Figure 3, the DPPH radical scavenging activity of pure CS film (21.4%) was significantly lower than that of CS-MTPITC-α-CD film (29.8%). The antioxidant potential of pure CS was predominantly ascribed to the free amino groups at the C-2 positions, which could react with DPPH radicals [53]. The enhanced antioxidant efficacy of CS-MTPITC-α-CD film might be due to the hydrogen atom of the methylene group in MTPITC transferring to the DPPH free radicals [54]. Thus, the introduction of MTPITC-α-CD could improve the antioxidant activity of pure CS film, which contributed to guarding oxidation-sensitive foodstuffs. A similarly improved antioxidant property was also reported when CS-based film was enhanced with CD-encapsulated other bioactive compounds [23,37].

### 3.4. Antibacterial Activities of CS and CS-MTPITC-α-CD Films

Previous evidence suggests that meat products are highly susceptible to being contaminated by *Salmonella*, which thereby leads to foodborne diseases and economic loss worldwide [55]. The antibacterial capacities of CS and CS-MTPITC-α-CD films were assessed by the disk diffusion assay using *S*. *typhimurium*. As displayed in Table 1, the inhibition zone diameter of the CS-MTPITC-α-CD film (14.7 ± 0.6 mm) against *S*. *typhimurium* after co-incubation for 12 h was notably higher than that of the CS film (7.3 ± 0.6 mm). However, the inhibition zone of the CS film disappeared after 24 h treatment, while that of the CS-MTPITC-α-CD film was 11.7 ± 0.5 mm. These data suggest that the effective antibacterial activity of the CS-MTPITC-α-CD film still remained after co-incubation for 24 h. The effective antibacterial potential of the fabricated composite film might be related to the electrophilic activity of the thiocyanate group in MTPITC, which binds to sulfhydryl groups of certain enzymes in bacteria to further interfere with the metabolic and biosynthetic processes [56]. Moreover, the structure–activity studies also illustrated that the methylthio group of MTPITC also plays a pivotal role in suppressing bacterial activity [16,17]. The data in the current study confirmed that the introduction of MTPITC-α-CD to the CS polymer chain imparted CS with an enhanced and sustainable antibacterial efficacy. Similar antibacterial behavior was also reported in the research using CS film enriched with the octyl gallate-β-CD inclusion complex [43]. 

### 3.5. Application of the CS and CS-MTPITC-α-CD Films on Fresh Chicken Meat

**Figure 4:**
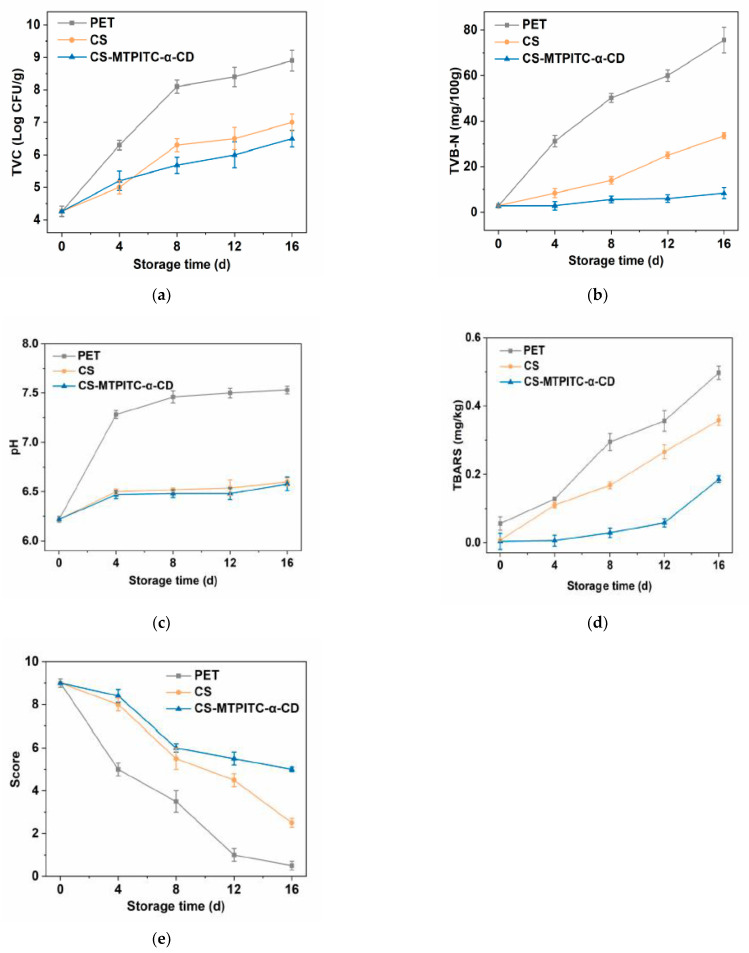
Quality index analysis of chicken samples wrapped with CS and CS−MTPITC−α−CD films during storage. (**a**) TVC; (**b**) TVB-N; (**c**) pH; (**d**) TBARS; (**e**) sensory evaluation.

Considering that the CS-MTPITC-α-CD composite film showed excellent antimicrobial potential in repressing the *S. typhimurium* growth, its inhibitory effect on the TVC of chicken meat under 4 °C storage was studied. The initial TVC of the meat sample was around 4.3 Log CFU/g, indicating an acceptable microbial quality of fresh chicken meat (Figure 4a). The TVC levels of all wrapped groups rose with the prolonged storage time, especially the PET-treated group showing the fastest increment rate. The PET film-wrapped chicken sample reached 8.1 Log CFU/g on day 8, which was over the threshold of fresh meat (7.0 Log CFU/g), indicating meat spoilage [57]. The CS film-wrapped chicken sample reached 7.0 Log CFU/g on the last day, whereas the CS-MTPITC-α-CD film-treated meat sample (6.5 Log CFU/g) remained lower than the detection limit, indicating that the composite film effectively prolonged the preservation period of the chicken meat to up to 16 d. Sun et al. [58] examined the influence of CS film combined with apple polyphenols on grass carp, demonstrating a notable reduction in microorganism proliferation during 8 d of refrigerated storage. Yaghoubi et al. [59] corroborated that the CS coating containing *Artemisia fragrans* essential oil showed excellent antibacterial efficacy against TVC proliferation in chicken fillets at 4 °C and extended the shelf life to 12 d. Taken together, CS-based packaging materials incorporated with antibacterial agents have the potential to be applied for preventing microorganism growth in fresh foodstuffs under refrigerated conditions.

The TVB-N value, derived from volatile nitrogenous compounds generated by bacteria-induced protein and nitrogen-containing compound degradation, is a principal parameter for evaluating the freshness of different meats [60]. The TVB-N value of a fresh meat sample was stipulated as 15 mg/100 g [61]. As displayed in Figure 4b, the PET film group dramatically increased and exceeded the limit within 4 d, whereas the CS film group presented a much slower increase, which reached the limit after 8 d of storage. By contrast, the TVB-N value in the CS-MTPITC-α-CD film group was only 8.4 mg/100g on day 16, indicating that the composite film had a superb suppressing effect against TVB-N formations in chicken meat. Similarly, Hu et al. [62] demonstrated that the CS film containing the antibacterial agent chlorogenic acid exhibited a more favorable suppressive effect on the TVB-N values in shrimps when compared with applying CS film alone. Shahbazi et al. [63] revealed a repressive effect on the TVB-N generation of fish fillets coated with CS containing *Mentha spicata* essential oil and zinc oxide nanoparticles. They ascribed this finding to either the inhibition of bacterial growth or the lower efficacy of bacteria-induced oxidative deamination of nitrogen substances. 

Figure 4c depicts the pH changes in the test film-wrapped chicken meat samples. The increment of pH in the PET film group was the most rapid, ranging from 6.2 to 7.5 during the whole storage period. However, the pH in the chicken meat wrapped with the CS or CS-MTPITC-α-CD film presented a milder increase, with the value reaching around 6.6 on the last day of the refrigerated period. This level of pH is the result of the generation of alkaline substances, which comes from protein degradation induced by enzymic oxidation or microorganisms [64,65]. Therefore, a retarded pH level might be ascribed to the reduced TVB-N substances in chicken meats wrapped with CS-based films (Figure 4b). Similarly, a previous study also revealed that the antioxidant and antibacterial potentials of CS film containing polyphenols were beneficial to decreasing the TVB-N accumulation, which in turn delayed the increase in pH in fish fillets [58]. 

The TBARS derived from the lipid peroxidation products was examined to evaluate the lipid oxidation extent of test meat samples [66]. The TBARS values in all groups increased with the storage time (Figure 4d), possibly due to the unsaturated fatty acid oxidation [67]. The TBARS in the PET film group displayed the greatest increment with 0.5 mg/kg on day 16, followed by the CS film with nearly 0.4 mg/kg on the last storage day. By comparison, the value of the chicken meat sample wrapped with CS-MTPITC-α-CD film showed a significantly slower growth tendency throughout the whole storage period, with approximately 0.2 mg/kg at day 16. This might be related to the improved antioxidant capacity of the CS-MTPITC-α-CD film as demonstrated in Figure 3, which helped to reduce or prevent lipid oxidation occurrence. Li et al. [42] also proposed that the application of the CS-based film containing dihydromyricetin-hydroxypropyl-β-cyclodextrin complex on fried meatballs notably repressed the TBARS value increase during a seven-day storage compared to the use of pure plastic film. In addition, Yong et al. [68] suggested that CS/dialdehyde starch- (-)-epicatechin gallate composite film possessing an excellent oxygen barrier property and antioxidant capacity exhibited the best efficacy in suppressing the increase in the TBARS level in sunflower seed oil. Jiang et al. [69] demonstrated that wrapping with CS-based film containing lemon essential oil was capable of repressing the TBARS increment in grass carp, which was related to both the antioxidant activity of the added bioactive substance and its suppression of the oxygen permeability. Thus, we speculated that except for the enhanced antioxidant activity, the oxygen barrier capacity might also be the reason that the CS-MTPITC-α-CD film effectively inhibited lipid oxidation in chicken meat samples.

The results of the organoleptic property assessments of chicken meat wrapped with PET film, CS film, and CS-MTPITC-α-CD film are presented in Figure 4e. All chicken meat samples were evaluated based on a 10-point hedonic scale, in which a score lower than 5 was considered unacceptable for consumers. As a whole, the results indicated that the score of the chicken sample coated with the CS-MTPITC-α-CD film was obviously superior to those packaged with the PET or CS films, with a score of 5 on day 16. These findings suggested that packaging with the CS-MTPITC-α-CD film could maintain meat quality to an acceptable degree during low-temperature storage (4 °C). The reasons for this might be ascribed to a combination of improved functional properties. Firstly, the lower light transmittance (Figure 2e) was able to prevent meat spoilage arising from light exposure-induced oxidation [70]. Secondly, the favorable antibacterial capacity (Figure 4a) could effectively reduce the adverse influence brought by microorganisms on meats [71]. Collectively, the results of the present study show that the application of the CS-MTPITC-α-CD film on chicken meat was able to reduce nutritional and quality losses during storage. The study by Photisarattana et al. [72] also revealed that packaging pork meat with films containing ZnO could reduce microbial growth and lipid oxidation, extending the shelf-life of pork meat samples. Moreover, recent studies have proposed that the addition of active substances produces functional active packaging, which preserves several quality changes and microbial growth in food products, replacing the direct addition of preservatives into foods [73,74,75].

## 4. Conclusions

In the current study, the CS-based film enriched with a MTPITC-α-CD inclusion complex was prepared and its antibacterial and preservative potential were evaluated using fresh chicken meat. MTPITC-α-CD was completely embedded into the CS polymeric matrix by physical forces. Furthermore, the UV-vis light barrier capacity and the antioxidant ability of the pure CS film were remarkably enhanced by the addition of MTPITC-α-CD. The CS-MTPITC-α-CD film displayed an effective and sustainable antibacterial capacity against *S*. *typhimurium*. The CS-MTPITC-α-CD film could effectively inhibit microorganism growth, suppress the increments of TVB-N and pH, prevent lipid oxidation, and maintain the sensory properties, prolonging the shelf-life of fresh chicken meat during low-temperature storage. Thus, CS-based film functionalized with a MTPITC-α-CD inclusion complex could be utilized as a packaging material for preserving the freshness and quality of chicken meat under refrigerated storage.

## Figures and Tables

**Table 1:** The diameters of the inhibition zones of the CS and CS-MTPITC-α-CD films against *S. typhimurium*.

Samples	Time	
	12 h	24 h
CS	7.3 ± 0.6 mm ^b^	0.0 ± 0.0 mm ^b^
CS-MTPITC−α-CD	14.7 ± 0.6 mm ^a^	11.7 ± 0.4 mm ^a^

Values with different letters in the same column indicate statistical significance at *p* < 0.05.

## Data Availability

The data will be shared on reasonable request to the corresponding author.
